# Influence of dielectric perfusate on pulsed-field ablation-induced haemolysis

**DOI:** 10.1093/europace/euag124

**Published:** 2026-05-19

**Authors:** Dishiwen Liu, Mei Yang, Kaigeng Hu, Yuntao Fu, Yuanjia Ke, Yajia Li, Xiaojian Long, Yixuan Luo, Qingyan Zhao

**Affiliations:** Department of Cardiology, Renmin Hospital of Wuhan University, 238 Jiefang Road, Wuchang District, Wuhan 430060, PR China; Cardiovascular Research Institute, Wuhan University, Wuhan 430060, PR China; Hubei Key Laboratory of Cardiology, Wuhan University, Wuhan 430060, PR China; Department of Cardiology, Renmin Hospital of Wuhan University, 238 Jiefang Road, Wuchang District, Wuhan 430060, PR China; Cardiovascular Research Institute, Wuhan University, Wuhan 430060, PR China; Hubei Key Laboratory of Cardiology, Wuhan University, Wuhan 430060, PR China; Department of Cardiology, Renmin Hospital of Wuhan University, 238 Jiefang Road, Wuchang District, Wuhan 430060, PR China; Cardiovascular Research Institute, Wuhan University, Wuhan 430060, PR China; Hubei Key Laboratory of Cardiology, Wuhan University, Wuhan 430060, PR China; Department of Cardiology, Renmin Hospital of Wuhan University, 238 Jiefang Road, Wuchang District, Wuhan 430060, PR China; Cardiovascular Research Institute, Wuhan University, Wuhan 430060, PR China; Hubei Key Laboratory of Cardiology, Wuhan University, Wuhan 430060, PR China; Department of Cardiology, Renmin Hospital of Wuhan University, 238 Jiefang Road, Wuchang District, Wuhan 430060, PR China; Cardiovascular Research Institute, Wuhan University, Wuhan 430060, PR China; Hubei Key Laboratory of Cardiology, Wuhan University, Wuhan 430060, PR China; Department of Cardiology, Renmin Hospital of Wuhan University, 238 Jiefang Road, Wuchang District, Wuhan 430060, PR China; Cardiovascular Research Institute, Wuhan University, Wuhan 430060, PR China; Hubei Key Laboratory of Cardiology, Wuhan University, Wuhan 430060, PR China; Department of Cardiology, Renmin Hospital of Wuhan University, 238 Jiefang Road, Wuchang District, Wuhan 430060, PR China; Cardiovascular Research Institute, Wuhan University, Wuhan 430060, PR China; Hubei Key Laboratory of Cardiology, Wuhan University, Wuhan 430060, PR China; Department of Cardiology, Renmin Hospital of Wuhan University, 238 Jiefang Road, Wuchang District, Wuhan 430060, PR China; Cardiovascular Research Institute, Wuhan University, Wuhan 430060, PR China; Hubei Key Laboratory of Cardiology, Wuhan University, Wuhan 430060, PR China; Department of Cardiology, Renmin Hospital of Wuhan University, 238 Jiefang Road, Wuchang District, Wuhan 430060, PR China; Cardiovascular Research Institute, Wuhan University, Wuhan 430060, PR China; Hubei Key Laboratory of Cardiology, Wuhan University, Wuhan 430060, PR China

**Keywords:** Pulsed-field ablation, Atrial Fibrillation, Intravascular Haemolysis, Free Haemoglobin

## Introduction

In recent years, pulsed-field ablation (PFA), an emerging non-thermal ablation technique, has been successfully applied in the treatment of atrial fibrillation. However, as clinical data accumulate, the technology has revealed a unique complication—intravascular haemolysis (IH).^1–4^ Current evidence suggests that factors such as blood volume, catheter–tissue contact,^5^ electric field strength,^6^ number of PFA application,^1^ and catheters from different manufacturers^7^ can all influence IH. However, to date, no studies have evaluated the impact of the perfusate on IH.

## Methods

### In vivo experimental procedure

**Figure 1:**
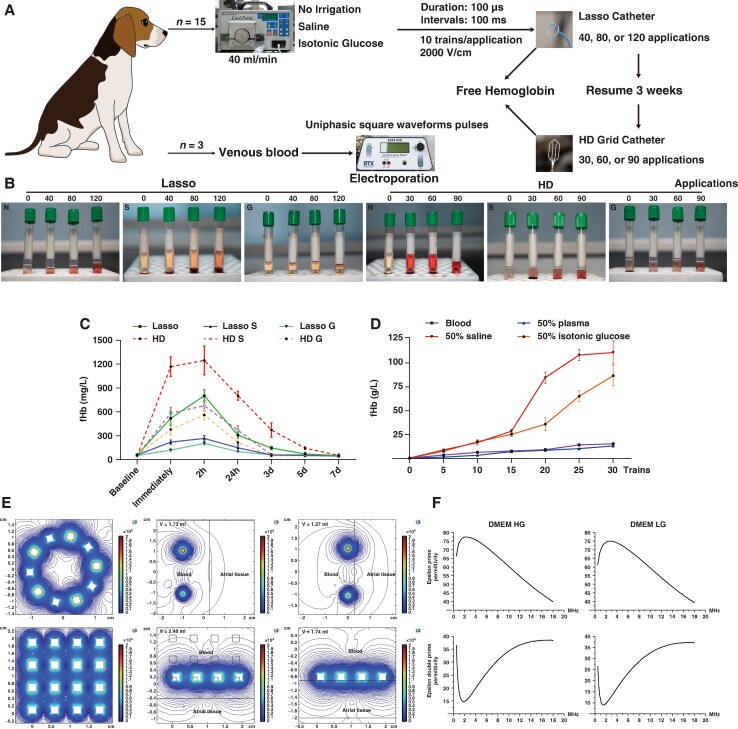
(*A*) Experimental Flow Chart; (*B*) Comparison of serum colour from pre-procedure to post-procedure in each group of *in vivo* experiments; Abbreviation: N, no irrigation; S, saline; G, isotonic glucose; (*C*) Trends in Free Haemoglobin (fHb) *in vivo* experiments; (*D*) Haemolysis In Vivo Erythrocytes with Different Dielectric Medium; (*E*) Lasso catheter and HD Grid catheter COMSOL simulation; V denotes the volume of blood exposed to the erythrocyte lethal threshold. The blue shading highlights the distribution of electric field that exceed erythrocyte lethal threshold. *(F*) Dielectric permittivity variations across commercial culture medium.

The beagles were divided into six groups: Non-irrigated Lasso (bipolar, St. Jude Medical, St. Paul, USA, Lasso group), Lasso with saline irrigation (Lasso S group), Lasso with isotonic glucose irrigation (Lasso G group), non-irrigated Advisor High Density Grid Mapping Catheter (bipolar, Abbott, Minneapolis, USA, HD group), HD with saline irrigation (HD S group), and HD with isotonic glucose irrigation (HD G group). Ablation was performed using the Lasso catheter. After 3 weeks of recovery, the same animals underwent a second ablation procedure using the HD catheter (*Figure 1A*).

### Ablation parameters

The ECM830 generator (BTX, USA) delivered uniphasic square waveforms pulses at 800 V (2000 V/cm). Each train consisted of pulses with a cumulative duration of 100 µs, delivered at 100 ms intervals. 10 trains and 1 s were applied per application. In the Lasso group, PFA was applied in the right atrium with 40, 80, or 120 applications. In the HD Grid group, 30, 60, or 90 applications were delivered. Irrigation was performed at 1 mL/min. 1 s before PFA delivery, high-pressure irrigation (40 mL/min) was initiated via the sheath or catheter and continued until the end of the PFA application, lasting approximately 2 s in total (*Figure 1A*).

### In vitro blood electroporation

The beagles’ plasma, saline, or isotonic glucose was mixed with equal volumes of whole blood. 500 μL mixture was transferred into an Electrode Cap Disposable Cuvette (4 mm gap, BTX, USA) for electroporation. Each train consisted of pulses with a cumulative duration of 100 µs, delivered at 100 ms intervals. 5 trains were applied per application.

## Results

### In vivo experiment

IH visibly intensified with the increasing number of PFA applications in all groups, with the HD group exhibiting the darkest coloration (*Figure 1B*). Free haemoglobin (fHb) levels peaked at 2 h post-PFA and decreased to baseline within 3–7 days, depending on the severity of IH. Irrigation, whether via the sheath or the catheter's integrated channel, significantly reduced fHb levels compared to non-irrigation groups (*Figure 1C*). In the Lasso S and G groups, isotonic glucose irrigation appeared more effective than saline in reducing IH. However, there was no signiﬁcant difference in the fHb levels between the Lasso S group and Lasso G group at 2 h post-PFA (*P* = 0.3822). For the multi-electrode HD Grid catheter, which induced the most severe IH, isotonic glucose irrigation nearly halved the fHb level at 2 h post-PFA (562.3 ± 63.9 vs. 1245.2 ± 181.3 mg/L, *P* < 0.0001) and was significantly superior to saline irrigation (562.3 ± 63.9 vs. 675.9 ± 63.1 mg/L, *P* = 0.0061).

### In vitro experiment

Whole blood did not exhibit the expected degree of haemolysis, which may be attributed to high cell density. Compared to whole blood, the 50% plasma group, with half the erythrocyte count, did not show a significant increase in fHb, and no statistical difference was observed between the two groups. Plasma demonstrated overwhelmingly superior erythrocyte protection compared to saline or isotonic glucose. Furthermore, after 20 trains, isotonic glucose provided significantly better protection than saline (83.4 ± 6.0 vs. 35.1 ± 6.5 g/L, *P* < 0.0001) (*Figure 1D*).

## Discussion

In this study, we verified that isotonic glucose irrigation significantly reduced IH. Vitro experiments confirmed that plasma was the most effective in counteracting haemolysis.

Clinically, some strategies address IH. Mohanty et al.^8^ showed that administering 2 L of fluid post-procedure significantly reduced haemoglobinuria and creatinine elevation. Recent studies have shown that saline irrigation can reduce haemolysis.^9^ Irrigation may reduce the occurrence of IH through several mechanisms. First, high-flow irrigation dilutes the blood surrounding the electric field, reducing the number of affected erythrocytes. Second, an interesting phenomenon occurs during pulsed-field delivery in the atrium. Pulses can transiently disrupt normal atrial contraction, slowing or stalling blood flow, particularly in paroxysmal atrial fibrillation patients in sinus rhythm. Adequate irrigation increases flow velocity, helping to swiftly carry erythrocytes away from the field zone.

Almost all currently published literature on IH can be reasonably explained by simulation data from COMSOL (*Figure 1E*). In both *in vivo* experiments and simulated data, we observed that IH increases as the number of electrode pairs increases. We speculate that electrodes in close proximity interfere with one another, resulting in a higher electric field strength. However, there is very little research on perfusate. Yuan et al.^10^ reported the interesting findings that Gd-DTPA reduces haemolysis during PFA, attributing the protective effect to its ability to stiffen erythrocyte membranes. However, Gd-DTPA itself carries a risk of contrast-induced nephropathy, limiting its widespread adoption. We posit that the plausible explanation for this effect is a change in the electrolyte's dielectric properties. We glimpsed the differing permittivity between high-glucose and normal-glucose culture media (*Figure 1F*). In addition, the conductivity of isotonic glucose is significantly lower than that of saline. The drop in electric field intensity in the blood is likely driven by the high impedance of the isotonic glucose. Surprisingly, *in vitro* experiments identified plasma as the optimal medium for reducing field strength. An alternative explanation for the aberrant *in vitro* findings should be considered. In particular, we believe the results may be explained by the absence of specific ions or metabolic substrates in saline and isotonic glucose compared with plasma. Even when isotonicity is preserved, erythrocytes can undergo haemolysis if essential components required for membrane stability, volume regulation, or cellular metabolism are lacking.

## Conclusion

Sheath-based irrigation with isotonic glucose can substantially reduce IH. Further development of catheters with irrigation function, coupled with a plasma-mimicking perfusate, holds promise for ultimately resolving the issue of IH.

## Data Availability

All supporting data of the present study are available in the article.
